# Clinicopathologic models predicting non‐sentinel lymph node metastasis in cutaneous melanoma patients: Are they useful for patients with a single positive sentinel node?

**DOI:** 10.1002/jso.26736

**Published:** 2021-11-04

**Authors:** Barbara Rentroia‐Pacheco, Félicia J. Tjien‐Fooh, Enrica Quattrocchi, Ajdin Kobic, Renske Wever, Domenico Bellomo, Alexander Meves, Tina J. Hieken

**Affiliations:** ^1^ Division of Bioinformatics SkylineDx B.V. Rotterdam The Netherlands; ^2^ Department of Dermatology Mayo Clinic Rochester Minnesota USA; ^3^ Department of Biochemistry and Molecular Biology Mayo Clinic Rochester Minnesota USA; ^4^ Division of Breast and Melanoma Surgical Oncology, Department of Surgery Mayo Clinic Rochester Minnesota USA

**Keywords:** melanoma, non‐sentinel lymph node, sentinel lymph node

## Abstract

**Background and Objectives:**

Of clinically node‐negative (cN0) cutaneous melanoma patients with sentinel lymph node (SLN) metastasis, between 10% and 30% harbor additional metastases in non‐sentinel lymph nodes (NSLNs). Approximately 80% of SLN‐positive patients have a single positive SLN.

**Methods:**

To assess whether state‐of‐the‐art clinicopathologic models predicting NSLN metastasis had adequate performance, we studied a single‐institution cohort of 143 patients with cN0 SLN‐positive primary melanoma who underwent subsequent completion lymph node dissection. We used sensitivity (SE) and positive predictive value (PPV) to characterize the ability of the models to identify patients at high risk for NSLN disease.

**Results:**

Across Stage III patients, all clinicopathologic models tested had comparable performances. The best performing model identified 52% of NSLN‐positive patients (SE = 52%, PPV = 37%). However, for the single SLN‐positive subgroup (78% of cohort), none of the models identified high‐risk patients (SE > 20%, PPV > 20%) irrespective of the chosen probability threshold used to define the binary risk labels. Thus, we designed a new model to identify high‐risk patients with a single positive SLN, which achieved a sensitivity of 49% (PPV = 26%).

**Conclusion:**

For the largest SLN‐positive subgroup, those with a single positive SLN, current model performance is inadequate. New approaches are needed to better estimate nodal disease burden of these patients.

## 1 INTRODUCTION

Clinically node‐negative cutaneous melanoma patients with a positive sentinel lymph node (SLN) are no longer routinely treated with immediate completion lymph node dissection (CLND). Both the large MSLT‐II and smaller DeCOG studies demonstrated that immediate CLND in SLN‐positive patients enrolled in these trials did not significantly improve melanoma‐specific survival compared to patients in the active surveillance group, who were offered CLND only when regional recurrence was identified.^1^, ^2^, ^3^ Consequently, and following a trend even pre‐dating the publication of these trial results, most SLN‐positive patients are managed with active surveillance^2^ as it has been clear for decades that only 10%–30% of clinically node‐negative (cN0) SLN‐positive melanoma patients will have additional metastatic disease in non‐sentinel lymph nodes (NSLNs) at CLND.^3^ As there is no widely accepted algorithm to predict NSLN status, there is a growing unmet clinical need to stratify cN0 SLN‐positive patients into a high‐risk group, which might be managed with active surveillance, and/or systemic therapies, and a low‐risk group, which might forgo not only additional surgery and systemic therapy, but nodal basin surveillance altogether.

Among the cN0 SLN‐positive patients, approximately 80% have only a single positive SLN.^4^ These patients are considered the lowest risk group among Stage III melanoma patients. However, still, one out of six patients will have additional metastases in the NSLN and will relapse in the at‐risk basin over time.^5^ Therefore, a reliable tool that supports the identification of single SLN‐positive patients would be of clinical value.

In recent years, several clinicopathologic models have been developed to predict NSLN positivity in SLN‐positive patients,^6^, ^7^, ^8^, ^9^ and some have been validated in external independent cohorts.^10^ However, only one of these models was designed explicitly for the subset of patients with a single positive SLN. The performance of the other models has not yet been assessed for this patient group. As a result, the utility of these other models for the majority of cN0 SLN‐positive melanoma patients remains unknown.

In this study, we sought to assess the ability of existing clinicopathologic models,^6^, ^7^, ^8^, ^9^ and of a newly developed clinicopathologic model to identify SLN‐positive melanoma patients with an increased risk for NSLN positivity. Specifically, we investigated the performance of these clinicopathologic models in predicting NSLN positivity for patients with a single positive SLN.

## 2 MATERIALS AND METHODS

### 2.1 Patient cohort

We retrospectively assembled a cohort of 200 patients treated at Mayo Clinic tertiary care centers in Minnesota, Arizona, or Florida between 2004 and 2017. This cohort represents a Stage III patient subset of a larger cohort previously described.^11^ All patients underwent lymphatic mapping with imaging and had a sentinel lymph node biopsy (SLNB) within 90 days of their primary melanoma diagnosis. Of these SLN‐positive patients, 153 underwent a CLND also within 90 days of their primary diagnostic biopsy. Ten patients were excluded for lack of available biopsy material. Thus, the total number of patients included was 143. The human investigations performed in this study were completed after approval by the Mayo Clinic Institutional Review Board and in accordance with the requirements of the Department of Health and Human Services, where appropriate.

### 2.2 Statistical methods

The probability of NSLN positivity was estimated using a logistic regression model. Specifically, we used LASSO regression,^12^ a regularized logistic regression that reduces the number of predictors for increased model interpretability. Models were built in R version 3.6.1 (R Foundation for Statistical Computing)^13^ using the *glmnet* package (v3.0.2).^14^ To reduce the number of predictors considered in our model (given the limited size of our cohort), we only included continuous clinicopathologic (CP) variables (exploiting the fact that CP variables are highly correlated among each other, therefore, we do not need all of them): age, number of positive SLN, Breslow thickness, mitotic rate, and the diameter of the largest SLN metastasis. We also log‐transformed the values of the last three variables, using a pseudo‐count of 0.01, to decrease the influence of outlier observations. To avoid potential differences between clinicopathologic practices, the number of positive SLN was used in the model as a binary variable (one or more than one SLN). Missing values were replaced by the median value of the corresponding variable across all patients, assuming that the values are missing at random. The dimension of the largest lymph node metastasis of patients with isolated tumor cells or a diameter less than 0.1 was set to be 0.01 and 0.099 mm, respectively.

### 2.3 Performance evaluation

Our new model was designed and evaluated using a repeated cross‐validation training/validation scheme, namely the double loop cross‐validation (DLCV), to ensure a form of internal validation method.^15^ DLCV efficiently separates feature selection and model optimization, which occur in the inner loop, from the model evaluation in the outer loop. As a result, reliable estimates of model performance on unseen patients can be obtained. We used ten inner folds for optimization of model parameters and five outer folds to evaluate the models. The procedure was repeated ten times. We optimized the clinicopathologic model by finding the combination of *λ* parameter that minimizes deviance across the inner test folds. We explored all possible combinations resulting from *glmnet*'s default regularization path for *λ* values.

Cross‐validated AUCs of our clinicopathologic model were determined by averaging outer loop AUCs using the R package *cvAUC* (v1.1.0) and then averaging these estimates over ten repetitions. Cutoff‐specific metrics were computed by concatenating true positives (TP), true negatives (TN), false positives (FP), and false negatives (FN) across outer folds of the same double loop and then calculating the performance metrics. These estimates were then averaged over 10 repetitions. Confidence intervals (CIs) of the average AUCs and other performance metrics were determined using the t‐distribution. CIs for the AUC of clinicopathologic models from the literature were obtained with the DeLong method.

The discriminative ability of all models was evaluated using widely used measures: sensitivity (SE), specificity (SP), negative predictive value (NPV), positive predictive value (PPV), with a corresponding 95% Clopper–Pearson CI^16^; and area under the receiver operating characteristic curve (AUC). We aimed to optimize a model for sensitivity and PPV.

### 2.4 Probability threshold for binary risk labels

Our primary goal was to identify patients at high risk of NSLN positivity correctly. Therefore, the output probabilities of the newly developed clinicopathologic model were converted into risk labels by setting a probability threshold so that patients with probabilities higher than the threshold are deemed high risk, and those with probabilities lower than the threshold are deemed low risk. This threshold was defined to maximize the F1‐measure during training. The F1‐measure^17^ is the harmonic mean of our target metrics (SE and PPV) and is expressed as $2\times \frac{\text{SE}\times \text{PPV}}{\text{SE}+\text{PPV}}$. By maximizing this measure, we aimed to find a probability threshold that would lead to a good tradeoff between our target metrics. We defined two different thresholds during DLCV: one optimized in the entire cohort and another optimized in patients with a single positive node only.

### 2.5 Comparison with publicly available models

The newly developed model was compared with four publicly available models that predict NSLN positivity via a nomogram or a scoring system developed by Gershenwald et al.,^8^ Bhutiani et al.,^9^ the N‐SNORE scoring system,^6^ and the nomogram developed by Bertolli et al.^7^ (Table 1). These models were selected because they were externally validated, and their input variables were available in our cohort. The Bhutiani model^9^ is the only model specifically designed for patients with a single positive SLN. The added value of all models was further assessed relative to two simple rules based on SLN tumor burden variables: number of positive SLNs (single vs. more than one) or diameter of largest SLN metastasis (≤1 mm vs. >1 mm). These are referred to as “Positive SLN rule” and “1 mm rule,” respectively. These models were implemented in R to calculate the risk score for each patient in our cohort. Patients were classified as low or high risk based on the probability threshold recommended in the original publications (for the nomogram) or the score corresponding to the highest possible risk category (for scoring systems). Patients with missing input variables were excluded from the analysis of the corresponding model.

**Table 1:** Characteristics of published models assessed in our cohort

Model	Variables used in the model	Type	Intended use population
Positive SLN rule	Positive SLN (single positive SLN or multiple)	Scoring system	Stage III
1 mm rule	Largest SLN diameter (≤1 mm or >1 mm)	Scoring system	Stage III
Gershenwald et al.^8^	Breslow thickness	Scoring system	Stage III
	Largest SLN diameter		
	Number SLN removed		
N‐SNORE^6^	Sex	Scoring system	Stage III
	Regression		
	% Positive SN		
	Perinodal lymphatic invasion		
	Largest SLN diameter		
Bertolli et al.^7^	Breslow thickness	Logistic regression model	Stage III
	Largest SLN diameter		
	Positive SLN		
Bhutiani et al.^9^	Multifocal microanatomical location of SLN tumor deposits	Logistic regression model	Stage III with one positive SLN
	Age		
	Largest SLN diameter		

Abbreviation: SLN, sentinel lymph node.

## 3 RESULTS

### 3.1 Study population

Out of the 143 patients included in this study, 25 (17%) had metastatic disease in at least one NSLN. Univariate analysis showed that age, Breslow thickness, number of positive SLNs, and the greatest linear dimension of the lymph node metastasis were significantly associated with NSLN positivity in this cohort (Table 2).

**Table 2:** Characteristics of sentinel node‐positive patients treated with completion lymph node dissection, stratified by non‐sentinel lymph node (NSLN) positivity

Characteristic		NSLN status		
	All patients	Negative (*n* = 118)	Positive (*n* = 25)	*p* *
**Gender**				
Female	57 (39.9%)	51 (43.2%)	6 (24.0%)	0.12
Male	86 (60.1%)	67 (56.8%)	19 (76.0%)	
**Age, years**	54 (38, 64)	50 (37, 60)	66 (57, 74)	**<0.01**
*Primary biopsy characteristics*				
**Biopsy location**				
Head/neck	22 (15.4%)	18 (15.3%)	4 (16.0%)	0.77
Trunk	62 (43.4%)	51 (43.2%)	11 (44.0%)	
Upper extremities	21 (14.7%)	19 (16.1%)	2 (8.0%)	
Lower extremities	24 (16.8%)	18 (15.3%)	6 (24.0%)	
Acral	14 (9.8%)	12 (10.2%)	2 (8.0%)	
**Breslow thickness, mm (IQR)**	2.25 (1.50, 3.20)	2.00 (1.41, 3.10)	3.10 (2.30, 4.00)	**<0.01**
**Clark level**				
II	1 (0.7%)	1 (0.8%)	0 (0.0%)	0.50
III	12 (8.4%)	11 (9.3%)	1 (4.0%)	
IV	120 (83.9%)	99 (83.9%)	21 (84.0%)	
V	8 (5.6%)	6 (5.1%)	2 (8.0%)	
Unknown	2 (1.4%)	1 (0.8%)	1 (4.0%)	
**Mitotic rate, per mm^2^, median? IQR?**				
	4.0 (2.0, 8.0)	4.0 (2.0, 7.0)	7.0 (3.0, 10.0)	0.05
Unknown	3 (2.1%)	3 (2.5%)	0 (0.0%)	
**Ulceration**				
Absent	86 (60.1%)	73 (61.9%)	13 (52.0%)	0.48
Present	56 (39.2%)	44 (37.3%)	12 (48.0%)	
Unknown	1 (0.7%)	1 (0.8%)	0 (0.0%)	
**Regression**				
Absent	116 (81.1%)	97 (82.2%)	19 (76.0%)	0.64
Present	5 (3.5%)	4 (3.4%)	1 (4.0%)	
Unknown	22 (15.4%)	17 (14.4%)	5 (20.0%)	
**Tumor‐infiltrating lymphocytes**				
Absent	31 (21.7%)	29 (24.6%)	2 (8.0%)	0.26
Non‐brisk	83 (58.0%)	66 (55.9%)	17 (68.0%)	
Brisk	12 (8.4%)	10 (8.5%)	2 (8.0%)	
Unknown	17 (11.9%)	13 (11.0%)	4 (16.0%)	
**Microsatellitosis**				
Absent	113 (79.0%)	94 (79.7%)	19 (76.0%)	0.51
Present	3 (2.1%)	2 (1.7%)	1 (4.0%)	
Unknown	27 (18.9%)	22 (18.6%)	5 (20.0%)	
**Angiolymphatic invasion**				
Absent	98 (68.5%)	82 (69.5%)	16 (64.0%)	0.26
Present	25 (17.5%)	18 (15.3%)	7 (28.0%)	
Unknown	20 (14.0%)	18 (15.3%)	2 (8.0%)	
**Histologic type**				
Superficial spreading	77 (53.8%)	66 (55.9%)	11 (44.0%)	0.53
Nodular	40 (28.0%)	30 (25.4%)	10 (40.0%)	
Other	19 (13.3%)	16 (13.6%)	3 (12.0%)	
Unknown	7 (4.9%)	6 (5.1%)	1 (4.0%)	
**In‐transit metastasis at diagnosis**				
Yes	4 (2.8%)	2 (1.7%)	2 (8.0%)	0.14
No	139 (97.2%)	116 (98.3%)	23 (92.0%)	
*Sentinel Lymph node biopsy characteristics*				
**SLNB finding**				
Positive		118 (100%)	25 (100%)	
Negative		0	0	
**Number of positive nodes**				
1	111 (77.6%)	97 (82.2%)	14 (56.0%)	**<0.01**
2	28 (19.6%)	20 (16.9%)	8 (32.0%)	
>2	4 (2.8%)	1 (0.8%)	3 (12.0%)	
**SLN metastatic disease burden**				
Isolated tumor cells	18 (12.6%)	17 (14.4%)	1 (4.0%)	0.09
Cell clusters < 0.1 mm (in greatest linear extent)	7 (4.9%)	7 (5.9%)	0 (0.0%)	
Cell clusters ≥ 0.1 mm, no extracapsular extension	104 (72.7%)	85 (72.0%)	19 (76.0%)	
Cell clusters ≥ 0.1 mm, with extracapsular extension	13 (9.1%)	8 (6.8%)	5 (20.0%)	
Unknown	1 (0.7%)	1 (0.8%)	0 (0.0%)	
**Dimension, largest lymph node metastasis (mm)**				
	1.05 (0.32, 3.00)	1.00 (0.20, 2.60)	3.00 (1.00, 5.60)	**<0.01**
Unknown	1 (0.7%)	1 (0.8%)	0 (0.0%)	

*Note*: Categorical and continuous variables are reported using total numbers (%) or median (interquartile range), respectively.

* *p*‐values of continuous and categorical variables were computed using the Wilcoxon rank‐sum test and the *χ*2 test (or Fisher exact test if expected cell counts <5), respectively.

### 3.2 Performance of clinicopathologic models in the prediction of NSLN positivity

**Figure 1:**
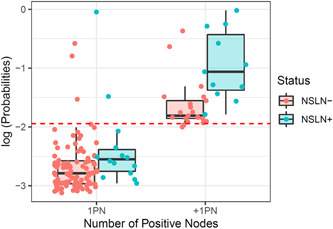
Output (log) probabilities from the Bertolli et al. model for patients with metastases in NSLN (NSLN+) and without metastases in NSLN (NSLN−). The dashed line indicates the probability threshold recommended by the authors to discriminate between high‐risk and low‐risk patients. This threshold is similar to dividing the patients according to whether they have a single positive node (1PN) or more than one (+1PN)

Our newly developed clinicopathologic model for NSLN positivity prediction included age, Breslow thickness (log), mitotic rate (log), largest SLN metastasis diameter (log), and the number of positive SLN (single vs. more than one node; see Table 3 for coefficients). The model predicted NSLN positivity with an AUC of 0.80 (95% CI = 0.79–0.80) and classified patients into two risk categories with an SE of 46% and PPV of 37% (Table 4). Compared to the SLN tumor burden‐based “two simple rules” and three previously published clinicopathologic models, the performance of our newly developed clinicopathologic model was similar to the Bertolli nomogram,^7^ which performed well (AUC = 0.76; 95% CI = 0.65–0.86; SE = 52%; PPV = 37%). Interestingly, while our model and the Bertolli nomogram had the highest overall discriminative ability, a rule‐of‐thumb that defines patients as high risk based on whether they have one or multiple positive SLN performed surprisingly well (SE = 44%; PPV = 34%). This stems from the fact that the risk for NSLN positivity is almost three times higher in patients with multiple positive SLN compared to patients with a single positive SLN (34% vs. 13%). The probability threshold defined by Bertolli et al.^7^ essentially separates patients based on whether they have a single or multiple positive SLNs (Figure 1).

**Table 3:** Logistic regression coefficients of the model developed in our cohort

	Clinicopathologic model coefficients
Intercept	−5.48
Age	0.05
Breslow thickness (log)	0.62
Mitotic rate (log)	0.02
SL positive nodes ≥2	1.33
Diameter of largest SLN metastasis (log)	0.41

Abbreviation: SLN, sentinel lymph node.

**Table 4:** Performance table for the entire cohort

Model	*N*	AUC (95% CI)	SP (95% CI)	SE (95% CI)	PPV (95% CI)	NPV (95% CI)
Positive SLN rule	143	0.63	82.2	44	34.4	87.4
		(0.53–0.74)	(74.1–88.6)	(24.4–65.1)	(18.6–53.2)	(79.7–92.9)
1 mm rule	142*	0.61	53.8	68	23.9	88.7
		(0.51–0.71)	(44.4–63.1)	(46.5–85.1)	(14.6–35.5)	(79.0–95.0)
Gershenwald et al.	142*	0.65	91.5	8.0	16.7	82.3
		(0.55–0.75)	(84.8–95.8)	(1.0–26.0)	(2.1–48.4)	(74.6–88.4)
N‐SNORE	120**	0.69	97.0	15.0	50.0	85.1
		(0.55–0.84)	(91.5–99.4)	(3.2–37.9)	(11.8–88.2)	(77.2–91.1)
Bertolli et al.	142*	0.76	81.2	52.0	37.1	88.8
		(0.65–0.86)	(72.9–87.8)	(31.3–72.2)	(21.5–55.1)	(81.2–94.1)
Newly developed model	143	0.80	83.0	45.6	36.7	87.8
		(0.79–0.80)	(79.9–86.1)	(40.5–50.7)	(33.4–40.0)	(87.0–88.7)

*Note*: The performance of our newly developed clinicopathologic model, two heuristic rules, and three publicly available clinicopathologic models have been characterized by the area under the receiver operating characteristic curve (AUC), specificity (SP), sensitivity (SE), positive predictive value (PPV) and negative predictive value (NPV). Confidence intervals of publicly available models for AUC and other metrics were calculated using DeLong and Clopper–Pearson methods, respectively. Confidence intervals of the newly developed clinicopathologic model were computed in the double loop procedure. (*) One patient was excluded due to the unknown largest SLN diameter, (**) 22 patients were excluded due to unknown regression, and one due to unknown largest diameter.

### 3.3 Performance of clinicopathologic models in patients with a single positive SLN

**Figure 2:**
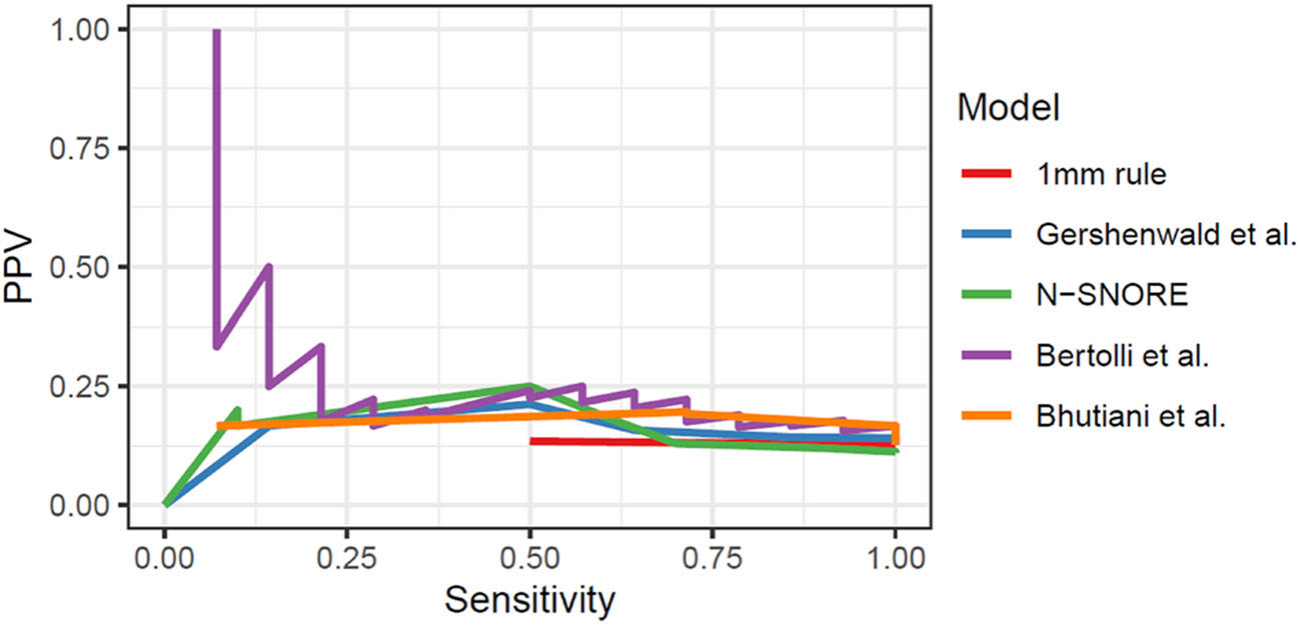
Positive predictive value (PPV) and sensitivity (SE) achieved by available clinic‐pathologic models among patients with a single positive SLN. Models evaluated were assigning patients to high‐risk or low‐risk groups based on a heuristic rule (“1 mm rule”), Gershenwald, N‐SNORE, Bertolli, and Bhutiani models. Curves are generated by varying the probability or score cutoff of the models and obtaining the corresponding PPV and sensitivity. SLN, sentinel lymph node

We further assessed the clinical relevance of the clinicopathologic models for patients with a single positive SLN, representing 78% (111 patients) of the entire cohort. The performance of all models decreased considerably in this patient group, and even the model specifically designed for this patient population, that is, the Bhutiani model^9^ (AUC = 0.68; 95% CI = 0.57–0.79), did not outperform the Bertolli model (AUC = 0.70; 95% CI = 0.56–0.83; Table 5). The poor sensitivity (<20%) achieved by most existing models illustrates that the recommended probability high‐risk score thresholds missed most NSLN positive patients with a single positive SLN. Moreover, varying the probability threshold for these models did not lead to a better tradeoff between sensitivity and PPV (Figure 2). Our newly developed clinicopathologic model with the probability threshold optimized for the entire cohort also achieved a low sensitivity in the single positive SLN patient group (23%). Therefore, we assessed a probability threshold optimized for patients with a single positive SLN during training. Our clinicopathologic model with an adjusted threshold led to a better tradeoff between the two metrics: PPV increased to 26% and sensitivity to 49%.

**Table 5:** Performance table for patients with a single positive SLN

Model	*N*	AUC (95% CI)	SP (95% CI)	SE (95% CI)	PPV (95% CI)	NPV (95% CI)
1 mm rule	110*	0.52	53.1	50.0	13.5	87.9
		(0.37–0.66)	(42.7–63.4)	(23–77)	(5.6–25.8)	(76.7–95.0)
Gershenwald et al.	110*	0.62	89.6	14.3	16.7	87.8
		(0.47–0.77)	(81.7–94.9)	(1.8–42.8)	(2.1–48.4)	(79.6–93.5)
N‐SNORE	91**	0.65	97.5	0	0	88.8
		(0.45–0.85)	(91.4–99.7)	(0.0–30.8)	(0.0–84.2)	(80.3–94.5)
Bertolli et al.	110*	0.70	96.9	14.3	40	88.6
		(0.56–0.83)	(91.1–99.4)	(1.8–42.8)	(5.3–85.3)	(80.9–94.0)
Bhutiani et al.	110*	0.68	94.8	7.1	16.7	87.5
		(0.57–0.79)	(88.3–98.3)	(0.2–33.9)	(0.4–64.1)	(79.6–93.2)
Newly developed model (probability threshold optimized on the entire cohort)	111	0.77	87.8	22.9	21.5	88.5
		(0.74–0.80)	(84.9–90.6)	(14.9–30.8)	(17.5–25.5)	(87.3–89.7)
Newly developed model (probability threshold optimized on one positive node patient)	111	0.77	79.7	49.3	26.4	91.4
		(0.74–0.80)	(77.7–81.8)	(42.3–56.3)	(24.0–28.7)	(90.1–92.7)

*Note*: The performance of our newly developed clinicopathologic model, one heuristic rule, and four publicly available clinicopathologic models have been characterized by the area under the receiver operating characteristic curve (AUC), specificity (SP), sensitivity (SE), positive predictive value (PPV) and negative predictive value (NPV). Confidence intervals of publicly available models for AUC and other metrics were calculated using DeLong and Clopper–Pearson methods, respectively. Confidence intervals of the newly developed clinicopathologic model were computed in the double loop procedure. (*) One patient was excluded due to the unknown largest SLN diameter, (**) 19 patients were excluded due to unknown regression, and one due to unknown largest diameter.

## 4 DISCUSSION

In this study, we assessed the ability of state‐of‐the‐art clinicopathologic models, designed to predict NSLN positivity, to identify high‐risk clinically node‐negative SLN‐positive melanoma patients who are most likely to benefit from additional therapeutic and/or intensive active surveillance strategies. We validated the performance of these clinicopathologic models in our cohort, and we found that the Bertolli model^7^ performed the best (AUC = 0.76; 95% CI = 0.65–0.86; SE= 52%, PPV = 37%), confirming the findings of another independent study.^10^ Additionally, we developed a new clinicopathologic model, which showed comparable performance (AUC = 0.80; 95% CI = 0.79–0.80), although it did not outperform the Bertolli model in terms of sensitivity and PPV. Together with multiple validation studies,^7^, ^10^, ^18^ the comparable discriminative abilities of the best performing models suggest an upper limit to the achievable performance, given the available clinicopathologic variables.

While the overall discriminative ability of models (as measured by the AUC) is relevant, assigning patients into a low or a high‐risk group (binary classification) may better inform clinical decisions, such as further surgical and/or systemic treatment and increased active surveillance. However, the risk stratification ability of clinicopathologic models was surprisingly similar to a simple binary rule based on SLN tumor burden, namely the number of positive SLNs (single vs. multiple). This is a direct consequence of the fact that the likelihood of NSLN positivity is considerably lower in patients with a single positive SLN: only 12% of patients with a single positive SLN had NSLN metastasis, compared to 34% of patients with multiple positive SLNs. We observed that the currently best performing model from Bertolli heavily relies on this association: its probability threshold essentially stratifies patients based on their number of positive SLNs. Therefore, it has limited applicability for patients with a single positive SLN.

This point deserves particular attention since the majority of SLN‐positive melanoma patients have a single positive SLN.^4^ For example, in our cohort, 78% of patients had only one positive SLN. As one in six patients with a single positive SLN will harbor NSLN metastases, and even though these patients are considered the lowest‐risk Stage III patients,^5^ we conducted additional subgroup analyses for this specific patient group. We observed that existing models lack the ability to correctly identify patients at high risk for NSLN positivity in this group—with sensitivities lower than 25% and PPV lower than 40%. This suggests that the models heavily rely on the information from the number of positive SLN and become less useful in the large subgroup of patients with one positive SLN. Interestingly, the only clinicopathologic model that was designed, or even assessed, in patients with a single positive SLN (the Bhutiani model)^9^ did not outperform the other clinicopathologic models, an observation which might be explained by the comparatively smaller size of its training cohort and the dichotomization of predictors.^19^

Low sensitivity and PPV could also result from using probability or score thresholds that are not designed for patients with a single positive SLN. As such, we optimized the probability threshold of the newly developed clinicopathologic model on this subgroup; however, this did not result in a reliable model for NSLN positivity prediction, as PPV remained similar and sensitivity increased only from 23% to 49%. Remarkably, changing the probability or score thresholds of all clinicopathologic models also failed to improve the tradeoff between SE and PPV. This would suggest that the performance is intrinsic to the models themselves rather than the chosen threshold.

The use of clinicopathologic models to predict NSLN positivity for clinically node‐negative SLN‐positive melanoma patients is promising; however, current models showed limited performance in our analysis. The success of the future clinicopathologic models will depend on their ability to complement the current staging system (N and T stages).

## 5 CONCLUSIONS

More work is needed to design models that can accurately predict NSLN positivity, specifically among patients with a single positive SLN. Optimally future studies should be based on large patient cohorts and explore the incorporation of predictors beyond the standard clinicopathologic variables. This approach may overcome the performance plateau that has been reached with the current models. To this end, the role of gene expression profiling of the index cutaneous melanoma, SLN metastasis, liquid biopsy, or afferent lymphatic channel fluid, in combination with clinicopathologic features, is a fertile avenue of exploration to improve melanoma risk stratification and tailor patient care.^11^, ^20^, ^21^

## CONFLICT OF INTERESTS

Dr. Hieken and Dr. Meves received research funding from SkylineDx. Dr. Hieken also received research funding from Genentech. Dr. Meves received honoraria from SkylineDx. Ms. Tjien‐Fooh, Ms. Wever, and Dr. Bellomo have equity stakes in SkylineDx. Ms. Rentroia‐Pacheco, Ms. Tjien‐Fooh, Ms. Wever, and Dr. Bellomo are or have been employees of SkylineDx. Dr. Bellomo and Dr. Meves report patents pending for gene signatures for predicting melanoma metastasis. All other authors report no conflicts of interest.

## SYNOPSIS

Approximately 80% of sentinel lymph node (SLN)‐positive patients have a single positive sentinel node and likely the lowest risk of harboring metastasis in non‐sentinel lymph nodes (NSLNs). Here, we assessed the performance of clinicopathologic models predicting NSLN metastasis on a cohort of 143 patients. We find that for the largest SLN‐positive subgroup, those with a single positive SLN, the performances of clinicopathologic models are inadequate and new approaches are needed if one seeks to better stratify risk among single SLN‐positive melanoma patients.

## Data Availability

The data that support the findings of this study are available from the corresponding author upon reasonable request.
